# Social Network Sites and Well-Being: Is it Only a Matter of Content?

**DOI:** 10.5334/irsp.736

**Published:** 2023-05-05

**Authors:** Alexandra Masciantonio, Maxime Résibois, Pierre Bouchat, David Bourguignon

**Affiliations:** 1 Maastricht University, NL; 2 CRéSaM (Centre de Référence en SantéMentale), BE; 3 University of Lorraine, FR

**Keywords:** well-being, social network sites, Facebook, Instagram, Twitter, content

## Abstract

Are social network sites harmful to our well-being? Despite the topicality of this question, the literature is still inconsistent. Possible reasons include the over-use of cross-sectional designs, the centration on Facebook, and the omission of the different ways of using these platforms. Two preregistered experimental studies were therefore conducted to investigate the effects of passive and active usages of Facebook, Instagram, and Twitter on subjective well-being. For both the first (N = 244) and the second (N = 164) study, the results did not yield any significant effects of the type of social network sites and their passive-active usages on subjective well-being. In contrast, surfing content was associated with subjective well-being in Study 2: the more positive the content was, the more life satisfaction increased, and the more the negative affect decreased. Further investigation of this research question will be necessary in larger samples, including longitudinal studies that could provide greater ecological validity while testing the effects of social network sites in the long-term. These findings are therefore to be taken with caution; above all, they open new avenues of research to understand the relationship between social network sites and subjective well-being.

## Introduction

Our lives are more and more affected by social network sites (SNSs). Facebook has almost 3 billion monthly active users (*Global Social Media Stats*, 2022). Although Facebook is the most used platform worldwide, there is an endlessly increasing number of other SNSs, such as Instagram and Twitter, which are among the 10 favorite SNSs in the world (*Global Social Media Stats*, 2022). Therefore, they are becoming a broad object of study for social psychologists: SNSs are our sidekicks for finding true love, acting collectively, or showing both antisocial and prosocial behaviors (Amichai-Hamburger, 2013). As influential as they can be, one question remains hotly debated in the literature: how SNSs impact our well-being?

### Social network sites and well-being

A lot of studies have been conducted these last decades, leading to seemingly inconsistent results: the effects of SNSs on well-being are sometimes beneficial (Kim & Lee, 2011; Valenzuela et al., 2009), sometimes damaging (Bevan et al., 2014; Lin et al., 2016). Meta-analyses have shown a general negative association between the SNSs use and well-being (Huang, 2017; Liu & Baumeister, 2016; Song et al., 2014; Yoon et al., 2019), albeit effect sizes are small, and associations depend on the indicator of mental health assessed (e.g., satisfaction with life, depression, loneliness, etc.).

Rather than relying on the general use of SNSs, some studies have therefore differentiated between an active usage (interacting with other users) and a passive one (consuming the content published by these users) (Burke et al., 2010; Frison & Eggermont, 2016; Shaw et al., 2015; Verduyn et al., 2015). The Active-Passive Model emphasizes that using SNSs actively leads to more social support and connectedness and in so doing, improves well-being (Verduyn et al., 2017). Conversely, using SNSs passively fosters upward social comparison and envy, worsening well-being. The Extended Active-Passive Model goes further by taking into account two additional characteristics of active use, reciprocity and communion, and two additional characteristics of passive use, self-relevance and content achievement (Verduyn et al., 2022). The Extended Active-Passive Model also highlights that user characteristics are essential to capture the complexity of the effects of passive and active usages on subjective well-being (Verduyn et al., 2022). In this regard, the meta-analysis of Liu et al. (2019) revealed that using SNSs for entertainment or for interacting with others is positively associated with well-being while using them for passively consuming SNSs’ content is negatively associated with well-being.

This literature offers a comprehensive framework for understanding the impacts of SNSs on wellbeing, but the methodological approaches to test it limit its scope. One major pitfall is the study design: most of the studies are cross-sectional—which does not allow for addressing the question of causality (Orben, 2020)—whereas less than 20% are experimental (Griffioen et al., 2020). In addition, due to its popularity, the focus of many studies has been on the Facebook platform (Valkenburg et al., 2021). For instance, of the 67 samples included in the meta-analysis of Huang (2017), 50 concerned Facebook and 16 concerned all SNSs combined.

### Cross-media perspective

This may be problematic, as all SNSs cannot be put in the same basket (Masciantonio et al., 2021). SNSs certainly differ in their affordances (O’Riordan et al., 2016), their users’ motivations (Alhabash & Ma, 2017), or their social contexts (Boczkowski et al., 2018). For example, Facebook is a bidirectional SNS, based on text and image. Users search both to keep in touch with their relatives and to present an idealized image of themselves (Nadkarni & Hofmann, 2012). Instagram is a unidirectional SNS, based on images. People use the platform to see what others share, to archive events of their life, to become popular, or to show their creativity (Sheldon & Bryant, 2016). Finally, Twitter is a unidirectional SNS, based on text. Twitter is also a well-known place for sharing and getting information (Johnson & Yang, 2009) and for mobilizing people (Park, 2013). In addition, the expression of negative emotions is perceived as more appropriate for Twitter and Facebook than for Instagram, although the expression of positive emotions is perceived as more appropriate for Instagram and Facebook than Twitter (Waterloo et al., 2018).

Due to these differences, the question remains whether the distinct effects of passive and active use can be generalized to all SNSs. For example, does passive use of Twitter—where negative emotions are more present—still lead users to compare themselves to people they perceived as having a better life than them?

### Differential impact of social network sites on well-being

Few studies have examined the relationship between other SNSs than Facebook and well-being. Among those, the results are overall contradictory. Two studies found that Instagram undermines well-being (Chae, 2018; Faelens et al., 2021), whereas the opposite was found in the study of Pittman and Reich (2016). One study found no significant association between Twitter use and well-being (Pittman & Reich, 2016), whereas another found a positive one (Chae, 2018). In addition to having contradictory results, these studies did not consider the distinct usages of SNSs. To our knowledge, only one study has investigated the impact of passively versus actively using various SNSs on well-being. Masciantonio et al. (2021) showed that passively using Facebook is negatively associated with well-being through upward social comparison. On the contrary, active usages of Instagram and Twitter were positively associated with well-being through social support. Surprisingly, passively using Twitter was negatively associated with upward social comparison, which in turn was negatively associated with well-being. Nevertheless, this study had one major limitation that needed to be addressed: its design was cross-sectional.

## Overview of the Present Studies

To fill the gap in the literature, we conducted two studies assessing the differential impact of various SNSs uses on well-being while using an experimental design. In these studies, we distinguished between passive and active usages while integrating two associated underlying mechanisms, namely social support and upward social comparison. To increase transparency and reproducibility in social media literature (Orben, 2020), all the data, scripts, and materials are available on the Open Science Framework. The two studies are also preregistered (see Open Practices section).

Participants in both studies were randomly assigned to passively or actively use Instagram, Facebook, or Twitter. Their subjective well-being was measured before and after the experimental manipulation. In Study 1, the time of use of the SNS was 10 minutes. In Study 2, the time of use was doubled. In addition, in Study 2, the content viewed by the participants and the device they used were controlled for.

The literature suggests that active use of SNSs is positively associated with well-being through social support, whereas for passive use the association is negative through upward social comparison (Verduyn et al., 2017). However, it appears that the specificities of Twitter—possibly the fact that posts are more negative—may be driving passive use of Twitter to be negatively associated with upward social comparison (Masciantonio et al., 2021). In doing so, 10 main hypotheses and 9 supplementary hypotheses have been preregistered. The complete set of hypotheses are available at the pre-registration links for Study 1 (https://doi.org/10.17605/OSF.IO/SE73R) and Study 2 (https://doi.org/10.17605/OSF.IO/S8UXP).

## Study 1

### Method

#### Participants

The required sample size of 228 participants was determined on G*Power for a repeated measures ANOVA with an alpha of 0.05, a power of 0.95 and an effect size of 0.15. To anticipate potential participants failing the seriousness check question, we targeted a 10% higher sample size. The study was conducted on the Prolific recruitment platform; participants received a small amount of money in exchange for their participation. To have a sample of active users of SNSs, we included participants only if they reported using Facebook, Instagram, and Twitter at least once a month. Besides, since the literature has primarily relied on student samples, non-students were targeted.

A total of 605 persons accessed our questionnaire, from which were removed those who did not fully complete the study (N = 321), did not give consent to participate in the study (N = 32), were students (N = 2), or did not follow the experimental instructions (N = 6).^1^ The final sample is thus composed of 244 participants. A sensitivity analysis was conducted to identify the minimum effect size that the study can reliably detect on G*Power (Perugini et al., 2018). For a sample size of 244 and an alpha level of 0.05 with 95% power, the study can detect a small effect size as expected (effect size f = 0.114).

Concerning socio-demographic information, the sample is composed of 127 men, 116 women, and 1 unknown. Participant’s ages ranged from 18 to 65 years old (M = 29.84, SD = 8.11). All were English native speakers and more than 70% of them were European. As said previously, no participants were students: 120 had a full-time job, 52 were unemployed, 39 had a part-time job, 10 were not in paid work (e.g., homemaker, retired, or disabled), and 23 were in another situation.

#### Pre-manipulation measures

Using the Qualtrics software, an online questionnaire was created in English. All participants signed an informed consent form before taking part in the study.

Before the experimental manipulation, participants responded to two questionnaires assessing baseline subjective well-being. First, cognitive subjective well-being was assessed through the Satisfaction with Life Scale (SWL; Diener et al., 1985). Second, affective subjective well-being was measured with the international Positive Affect and Negative Affect Schedule (PANAS) Short Form in 10 items (I-PANAS-SF; Thompson, 2007), which assumes the existence of two dimensions of affect: positive and negative. The three scales are reliable (Béland et al., 2017): McDonald Omega coefficient (ω) for SWL is 0.92, ω for positive affect is 0.74, and ω for negative affect is 0.82.

#### Experimental manipulation

The experimental manipulation consisted of a 3 (Type of SNS: Facebook vs. Instagram vs. Twitter) × 2 (Usage of the SNS: active *vs* passive) × 2 (Time: subjective well-being before using SNS versus subjective well-being after using SNS) factorial design. Participants were randomly assigned to one of the six experimental conditions using the Qualtrics randomization options. The instructions are presented in Table 1.

**Table 1:** Instructions for experimental conditions in Study 1.


EXPERIMENTAL CONDITION	INSTRUCTION

Active Facebook use (N = 41)	We are interested in the way you use Facebook to post and communicate with others. So, for the next 10 minutes, we ask that you try using Facebook only for direct communication—for example, updating your status; reacting, sharing, and commenting on your friends’ posts; sending and responding to private messages; etc. In addition, we ask that you only use Facebook for direct communication and refrain from browsing, such as scrolling your news feed; looking at your friends’ profiles; looking up information; etc. While you are communicating directly on Facebook, we ask that you refrain from clicking on any links that will lead to non-Facebook sites.

Passive Facebook use (N = 39)	We are interested in the way you use Facebook to watch or read others’ contents. So, for the next 10 minutes, we ask that you try using Facebook only for browsing (without liking or commenting on anything)—for example, scrolling your news feed; looking at your friends’ profiles; looking up information; etc. In addition, we ask that you only use Facebook for browsing and refrain from posting or communicating with others, such as updating your status; reacting, sharing, and commenting on your friends’ posts; sending and responding to private messages; etc. While you are browsing on Facebook, we ask that you refrain from clicking on any links that will lead to non-Facebook sites.

Active Instagram use (N = 41)	We are interested in the way you use Instagram to post and communicate with others. So, for the next 10 minutes, we ask that you try using Instagram only for direct communication—for example, posting photos or videos; liking and commenting on your followers’ posts; sending and responding to direct messages; etc. In addition, we ask that you only use Instagram for direct communication and refrain from browsing, such as scrolling your news feed; looking at your followers’ profiles; looking up for information; etc. While you are communicating directly on Instagram, we ask that you refrain from clicking on any links that will lead to non-Instagram sites.

Passive Instagram use (N = 43)	We are interested in the way you use Instagram to watch or read others’ contents. So, for the next 10 minutes, we ask that you try using Instagram only for browsing (without liking or commenting on anything)—for example, scrolling your news feed; looking at your followers’ profiles; looking up for information; etc. In addition, we ask that you only use Instagram for browsing and refrain from posting or communicating with others, such as posting photos or videos; liking and commenting on your followers’ posts; sending and responding to direct messages; etc. While you are browsing on Instagram, we ask that you refrain from clicking on any links that will lead to non-Instagram sites.

Active Twitter use (N = 39)	We are interested in the way you use Twitter to post and communicate with others. So, for the next 10 minutes, we ask that you try using Twitter only for direct communication—for example, posting a Tweet; liking, retweeting, and/or replying to a Tweet; sending and responding to direct messages; etc. In addition, we ask that you only use Twitter for direct communication and refrain from browsing, such as scrolling your news feed; looking at your followers’ profiles; looking up for information; etc. While you are communicating directly on Twitter, we ask that you refrain from clicking on any links that will lead to non-Twitter sites.

Passive Twitter use (N = 41)	We are interested in the way you use Twitter to watch or read others’ contents. So, for the next 10 minutes, we ask that you try using Twitter only for browsing (without liking or commenting on anything)—for example, scrolling your news feed; looking at your followers’ profiles; looking up for information; etc. In addition, we ask that you only use Twitter for browsing and refrain from posting or communicating with others, such as posting a Tweet; liking, retweeting, and/or replying to a Tweet; sending and responding to direct messages; etc. While you are browsing on Twitter, we ask that you refrain from clicking on any links that will lead to non-Twitter sites.


To ensure data validity, we implemented two compliance techniques. The first was the utilization of a countdown timer: participants could not continue with the questionnaire until the 10-min time limit had passed. Secondly, we used the seriousness check method (Aust et al., 2013). We asked participants ‘It would be very helpful if you could tell us whether you have seriously followed the [Facebook][Instagram][Twitter] use instruction so that we can use your answers for our scientific analysis? Be aware that your monetary compensation and your admission to future surveys will NOT be affected: your response will be without consequences for you.’ Participants who responded ‘No’ were excluded from all analyses.

#### Post-manipulation measures

After the experimental manipulation, participants responded again to the same measures of subjective well-being as presented in the pre-manipulation measures section (Diener et al., 1985; Thompson, 2007). Reliability is again satisfying (ω for SWL = 0.92; ω for positive affect = 0.79; ω for negative affect = 0.83). Additionally, we assessed the general frequency of use of Facebook, Instagram, and Twitter on a seven-point scale (never, rare, monthly, a few times per month, weekly, a few times per week, daily).

Finally, we asked participants to respond to several questions regarding the SNS they used. Social support on Facebook, Instagram, or Twitter was assessed with an adaptation of the four items scale proposed by Hofhuis et al. (2019). An example item is: ‘My [Facebook][Instagram][Twitter]/[friends][followers] really try to help me’ (ω = 0.88). Upward social comparison on Facebook, Instagram, or Twitter was measured with the four-item scale proposed by Lim and Yang (2019). A sample item is: ‘Many of my [friends][followers] on [Facebook][Instagram][Twitter] have a better life than me’ (ω = 0.81). Finally, one recent study showed that upward social comparison can improve—rather than decrease—well-being if it triggers inspirational motivation through benign envy (Meier et al., 2020). Taking this into account, we also measured benign and malicious envy for exploratory purposes. Following Lim and Yang (2019), benign envy was assessed with the single item ‘If I notice that another person is better than me on [Facebook][Instagram][Twitter], I try to improve myself,’ and malicious envy with the single item ‘When I see other people’s achievements on [Facebook][Instagram][Twitter], it makes me resent them.’

### Results

All analyses were conducted on R software version 4.1.3. An exploratory data analysis (EDA) is available on OSF (https://osf.io/9wybs/files/osfstorage/622f40cb46bf100cc9594f4a).^2^

#### Preliminary analyses

In addition to the two compliance techniques presented previously, we also measured the time participants spent on SNS during the experimental manipulation. We conducted an ANCOVA to check if this time differs according to the modalities of usage or the type of SNS. When controlling for age and gender, time did not differ regardless of whether participants used Facebook, Instagram, and Twitter, and whether it was passively or actively (for interaction effect, F(2, 235) = 0.035, p = 0.965, η^2^_G_ = 0.000).

#### Main analyses

To control the effects of age and gender, we used them as covariates in our analyses.^3^ For all components of subjective well-being (satisfaction with life, positive affect, and negative affect), we performed a 3 (Type of SNS: Facebook vs. Instagram vs. Twitter) × 2 (Usage of the SNS: active vs. passive) × 2 (Time: before using SNS vs. after using SNS) repeated measures ANCOVA.^4^

##### Satisfaction with life

Table 2 presents descriptive statistics for the satisfaction with life according to our experimental conditions.

**Table 2:** Descriptive statistics for satisfaction with life in Study 1.


SNS	USAGE	T_1_ SWLM(SD)	T_2_ SWLM(SD)

Facebook	Active	4.18 (1.41)	4.20 (1.46)

	Passive	4.27 (1.28)	4.07 (1.40)

Instagram	Active	4.06 (1.42)	3.98 (1.35)

	Passive	4.35 (1.20)	4.31 (1.22)

Twitter	Active	4.11 (1.37)	4.08 (1.47)

	Passive	3.77 (1.63)	3.80 (1.64)


**Figure 1:**
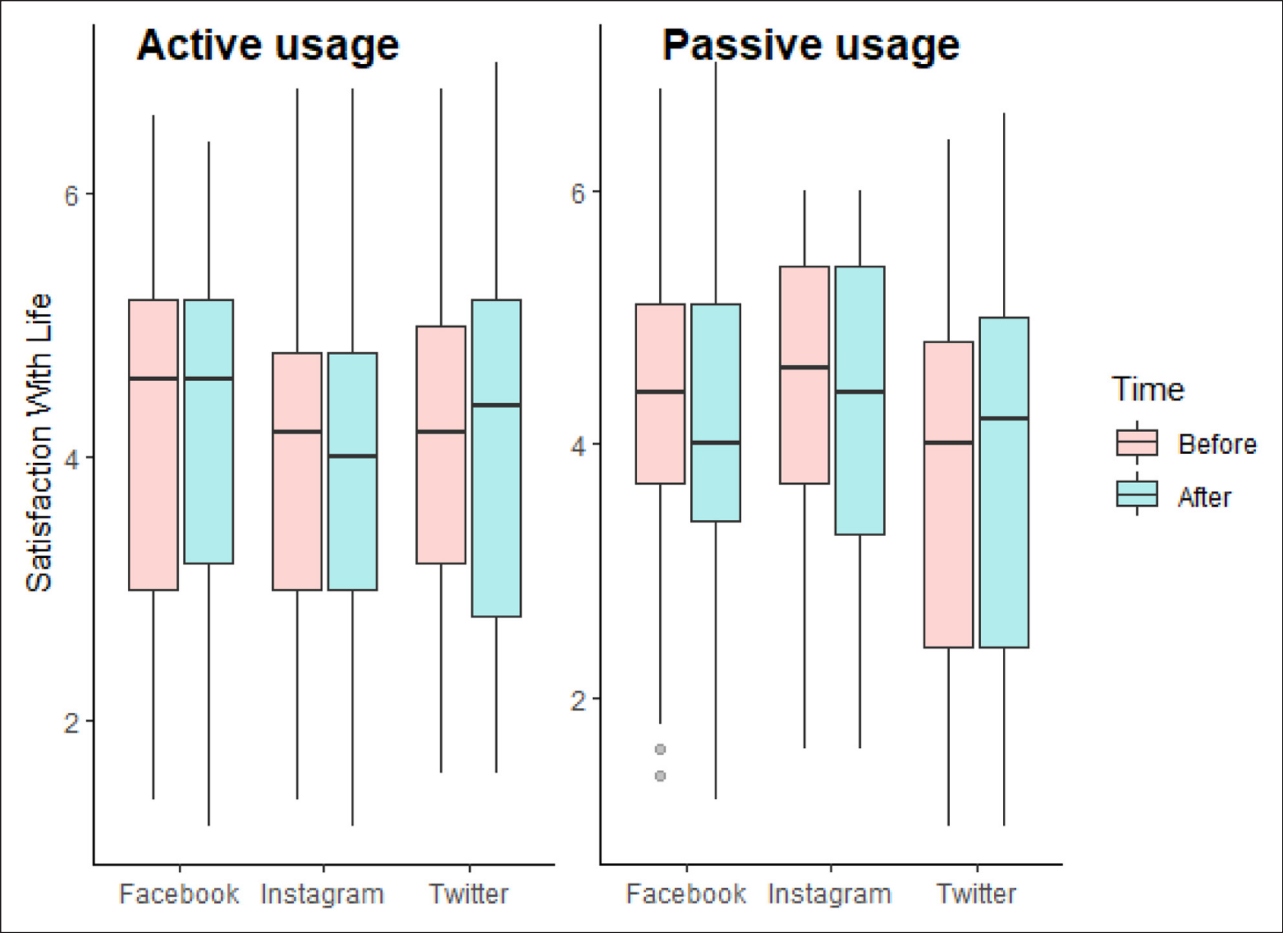
Boxplots of satisfaction with life according to the experimental conditions in Study 1.

As we can see in Figure 1, the repeated measures ANCOVA for satisfaction with life did not reveal any significant effect of our independent variables (for interaction effect, F(2, 235) = 1.845, p = 0.160, η^2^_G_ = 0.000). We however found an effect of covariates: satisfaction with life was statistically associated with participants’ age, F(1, 235) = 4.727, p = 0.031, η^2^_G_ = 0.019: age was positively associated with satisfaction with life at time 2 (r(242) = 0.159, p = 0.013).

Our results do not show a significant effect of passive and active usages of various SNSs on satisfaction with life. However, non-significant results cannot be understood as the absence of an effect (Quertemont, 2011). We, therefore, performed a repeated measures ANCOVA with an objective Bayesian approach (van den Bergh et al., 2020). The model-averaged results across matched models are presented in Table 3. The exclusion Bayes factors for models including our independent variables are between 1.46 and 13.69, signifying that our data provides anecdotal to substantial evidence in favor of H0 (Wetzels & Wagenmakers, 2012). Regarding our covariates, the data are more likely under the models including age (BF_excl_ = 0.43).

**Table 3:** Model-averaged results for satisfaction with life in Study 1.


EFFECTS	BF_EXCL_

Time	3.18

SNS	2.17

Usage	2.33

Age	0.43

Gender	1.20

Time*SNS	13.69

Time*Usage	6.03

SNS*Usage	1.46

Time*SNS*Usage	2.31


##### Positive affect

Descriptive statistics for the positive affect according to our experimental conditions are shown in Table 4.

**Table 4:** Descriptive statistics for positive affect in Study 1.


SNS	USAGE	T_1_ PA M(SD)	T_2_ PA M(SD)

Facebook	Active	2.93 (0.76)	2.94 (0.81)

	Passive	2.88 (0.82)	2.74 (0.91)

Instagram	Active	2.95 (0.70)	2.95 (0.86)

	Passive	2.83 (0.77)	2.77 (0.76)

Twitter	Active	3.11 (0.83)	2.97 (0.81)

	Passive	2.88 (0.70)	2.85 (0.73)


**Figure 2:**
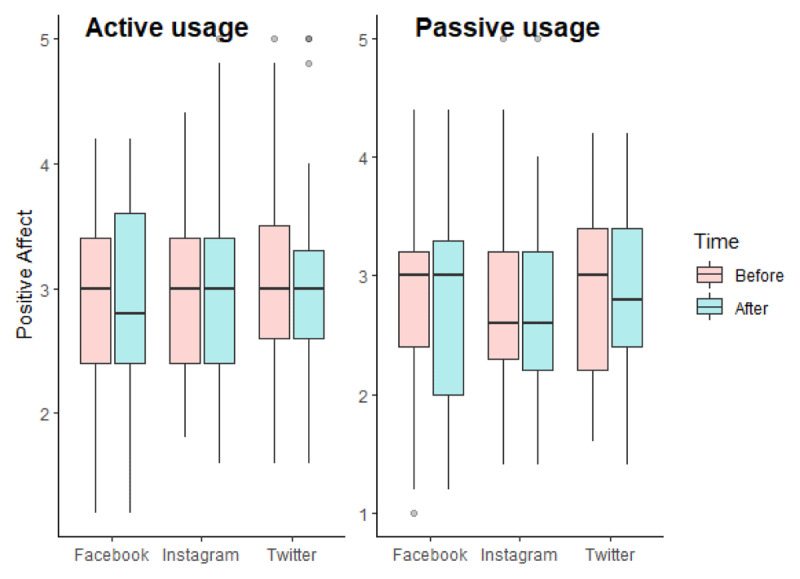
Boxplots of positive affect according to the experimental conditions in Study 1.

As we can see in Figure 2, the repeated measures ANCOVA for positive affect did not reveal any significant effect of our independent variables (for interaction effect, F(2, 235) = 0.968, p = 0.381, η^2^_G_ = 0.001). We however found an effect of covariates: there was a significant interaction between gender and time, F(1, 235) = 4.821, p = 0.029, η^2^_G_ = 0.002. We performed pairwise comparisons with the Bonferroni correction. We found no significant differences between men and women according to the time of measurement (p.adj for time 1 = 0.220; p.adj for time 2 = 0.682).

As previously, we performed a repeated measures ANCOVA with an objective Bayesian approach. The model-averaged results across matched models are presented in Table 5. The exclusion Bayes factors are superior to 1, signifying that our data provide anecdotal to substantial evidence in favor of H0.

**Table 5:** Model-averaged results for positive affect in Study 1.


EFFECTS	BF_EXCL_

Time	2.3

SNS	6.37

Usage	1.36

Age	2.06

Gender	3.21

Time*SNS	19.32

Time*Usage	6.83

SNS*Usage	4.77

Time*SNS*Usage	5.28


##### Negative affect

The descriptive statistics for the negative affect are presented in Table 6.

**Table 6:** Descriptive statistics for negative affect in Study 1.


SNS	USAGE	T_1_ NA M(SD)	T_2_ NA M(SD)

Facebook	Active	1.78 (0.87)	1.73 (0.85)

	Passive	1.41 (0.66)	1.46 (0.69)

Instagram	Active	1.61 (0.59)	1.49 (0.52)

	Passive	1.61 (0.77)	1.62 (0.69)

Twitter	Active	1.95 (0.76)	1.93 (0.87)

	Passive	1.80 (0.83)	1.80 (0.78)


**Figure 3:**
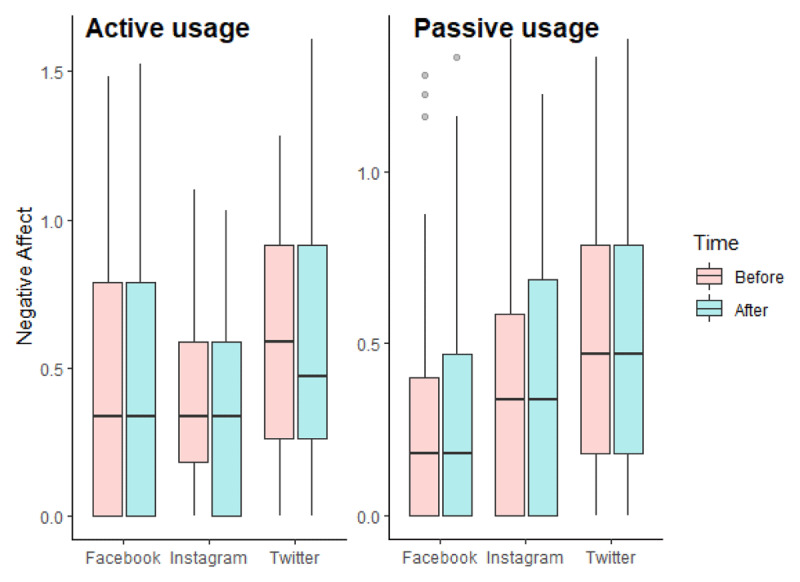
Boxplots of log-transformed negative affect according to the experimental conditions in Study 1.

The repeated measures ANCOVA revealed a main effect of the type of SNS (F(2, 235) = 5.122, p = 0.007, η^2^_G_ = 0.037) and an interaction effect between usage and time (F(1, 235) = 3.953, p = 0.048, η^2^_G_ = 0.002 (Figure 3). In addition, we found an effect of our two covariates: negative affect were statistically associated with participants’ age (F(1, 235) = 13.379, p = 0.000, η^2^_G_ = 0.048) and participants’ gender (F(1, 235) = 5.853, p = 0.016, η^2^_G_ = 0.022). For age, we found a negative association with negative affect at time 2: r(242) = –0.206, p = 0.001.

Regarding the main effect of the type of SNS, we performed Bonferroni’s multiple comparison tests. Results showed that negative affect were higher for participants who used Twitter than for participants who used Facebook or Instagram (p.adj for Facebook and Instagram = 1.000; p.adj for Facebook and Twitter = 0.001; p.adj for Instagram and Twitter = 0.002). However, the negative affect of participants in the Twitter conditions were already higher before the experimental manipulation (see Table 6). As such, this main effect of SNS could be explained as a coincidence from randomization. We also performed Bonferroni’s multiple comparison tests for exploring the interaction between usage and time. The analyses revealed that at time 1, participants experienced more negative affect in the active use condition (M = 1.78, SD = 0.75) than in the passive use condition (M = 1.61, SD = 0.77) (p.adj = 0.029). Again, this result offers little relevant information as this difference is found before our experimental manipulation.

Finally, we conducted a repeated measures ANCOVA with an objective Bayesian approach. The model-averaged results across matched models are presented in Table 7. The exclusion Bayes factors are in favor of H0 for models including the main effect of time, the interaction between time and SNS, the interaction between time, SNS, and usages, and the interaction between SNS and usage (BF_excl_ > 2). However, our data provide no evidence for H0, nor for the alternative hypothesis concerning models including the main effect of usage and the interaction between time and usage (BF_excl_ ≈ 1). Finally, the exclusion Bayes factors provide evidence in favor of the alternative hypothesis for including the type of SNS, age, and gender (BF_excl_ < 1).

**Table 7:** Model-averaged results for negative affect in Study 1.


EFFECTS	BF_EXCL_

Time	6.24

SNS	0.15

Usage	1.01

Age	0.01

Gender	0.21

Time*SNS	20.46

Time*Usage	1.01

SNS*Usage	2.69

Time*SNS*Usage	12.46


#### Exploratory analyses

##### Upward social comparison, benign envy, and malicious envy

We first compared the perception of upward social comparison, benign envy and malicious envy between Facebook, Instagram, and Twitter. We, therefore, performed three one-way ANCOVAs. After controlling for age and gender, ANCOVAs did not revealed any significant effect of the type of SNS on upward social comparison (F(2, 238) = 1.134, p = 0.323, η^2^_G_ = 0.009), benign envy (F(2, 238) = 2.092, p = 0.126, η^2^_G_ = 0.017), and malicious envy (F(2, 238) = 0.334, p = 0.716, η^2^_G_ = 0.003). We however found an effect of age (F(1, 238) = 10.011, p = 0.002, η^2^_G_ = 0.040) and gender (F(1, 238) = 5.887, p = 0.016, η^2^_G_ = 0.024) on upward social comparison.

We then looked for correlations between these three constructs. First, a positive association was found between upward social comparison and benign envy regardless of the type of SNS (r(78) = 0.424 for Facebook, p = 0.000; r(82) = 0.451 for Instagram, p = 0.000; r(78) = 0.395 for Twitter, p = 0.000). Second, a positive association was found between upward social comparison and malicious envy regardless of the type of SNS (r(78) = 0.398 for Facebook, p = 0.000; r(82) = 0.472 for Instagram, p = 0.000; r(78) = 0.526 for Twitter, p = 0.000). Third, a positive association was found between benign envy and malicious envy only for Facebook (r(78) = 0.349, p = 0.002) and Twitter (r(78) = 0.264, p = 0.018), but not for Instagram (r(82) = 0.193, p = 0.078).

Finally, no significant association was found between the frequency of SNS use and the perception of upward social comparison, benign envy, or malicious envy.

##### Social support

We compared the perception of social support between Facebook, Instagram, and Twitter. After controlling for age and gender, the one-way ANCOVA revealed an effect of the type of SNS, F(2, 238) = 10.364, p = 0.000, η^2^_G_ = 0.080. Bonferroni’s multiple comparison tests showed that social support on Facebook (M = 3.73, SD = 1.55) is statistically higher than social support on Instagram (M = 2.96, SD = 1.25) and on Twitter (M = 2.57, SD = 1.18).

We finally tested the association between the frequency of SNS use and the perception of social support. The more participants used Twitter, the more social support they perceived on the platform (r(78) = 0.355, p = 0.001). However, we found no significant association for Facebook (r(78) = 0.068, p = 0.550) and Instagram (r(82) = 0.159, p = 0.149).^5^

### Brief discussion

These results do not allow us to support the distinction between passive and active use: regardless of the usage, we did not find a significant impact of SNSs on subjective well-being (Verduyn et al., 2017). In addition, our results are also not consistent with the SNSs comparative approach: Facebook, Instagram, and Twitter did not have significantly distinct effects on subjective well-being (Masciantonio et al., 2021). Hence, our hypotheses cannot be supported. It should be noted, however, that Bayesian analyses did not always provide strong evidence in favor of the null hypothesis, suggesting that other factors may explain these non-significant results in the NHST approach.

The study does confirm some of the literature on SNSs. Indeed, upward social comparison was positively associated with benign envy and malicious envy (Lim & Yang, 2019; Meier et al., 2020). Besides, we found that social support is more important on Facebook than on Instagram or Twitter. This result can be explained when taking into account the cross-media perspective: Facebook consists of mutual friends, unlike Instagram and Twitter whose relationships are unidirectional and often with unknown people (Ellison et al., 2007). Interestingly, the more people use Twitter, the more social support they perceive on the platform, suggesting that the quality of the relationships on the platform increases over time.

Several methodological reasons can then explain the absence of significant results. First, the exposure time may have been too short to impact subjective well-being. Second, the literature is mainly based on young adults, whereas our sample was composed of non-student participants. One may hypothesize that the influential impact of SNSs may be greater for younger people as capacities to resist peer influence gradually increase with age, especially as age was found to influence social comparison (Albert et al., 2013). Third, SNSs surfing content was not considered. However, recent reviews suggested that what people see and publish during their utilization of SNSs can have an impact on their well-being (Meier & Reinecke, 2021; Valkenburg, 2022; Verduyn et al., 2022). To our knowledge, only one study investigated the effect of SNS content’s valence on subjective well-being (Choi & Kim, 2020), showing that the more positive the content on Instagram was, the more subjective well-being increased. However, only passive use of Instagram was assessed. Finally, the device that participants used to navigate on their SNSs was not taken into account, whereas it was found that smartphones can have a differential effect on well-being than computers or tablets for example (Fitz et al., 2019).

To address these limits, Study 2 was conducted, aiming at applying the same experimental protocol as Study 1, but with a longer SNS usage time (20 minutes, as in Sagioglou & Greitemeyer, 2014), on a younger population, and controlling for the surfing content as well as the device used.

## Study 2

### Method

#### Participants

As with Study 1, an a priori power analysis was determined on G*Power for a repeated measures ANOVA with an alpha of 0.05, a power of 0.95 and an effect size of 0.15. The required sample size was 228 participants, and we sought to collect a 10% larger sample.

A total of 395 participants were recruited during psychology lectures. Respondents were included if they reported using Facebook, Instagram, or Twitter at least once a month. As previously, we removed participants who did not fully complete the study (N = 228) or did not follow the experimental instructions (N = 3).^6^ The final sample consists of 164 participants. We conducted a sensitivity analysis on G*Power: for a sample size of 164 and an alpha-level of 0.05 with 95% power, the study can detect a small to medium effect size (effect size f = 0.18).^7^

Concerning socio-demographic information, the sample is composed of 136 women, 20 men, and 7 persons with another gender identity (one person did not give the information). Participants were all French students with age ranging from 17 to 35 years old (M = 19.91, SD = 2.95).^8^

#### Pre-manipulation measures

As in Study 1, the Qualtrics software was used. All participants signed an informed consent form. Subjective well-being—satisfaction with life (ω = 0.82), positive affect (ω = 0.62), and negative affect (ω = 0.78)—was assessed before the experimental manipulation (Diener et al., 1985; Thompson, 2007).

#### Experimental manipulation

The study design, the experimental instructions, and the compliance techniques are equivalent to those of Study 1: 28 participants were in the active Facebook use condition, 26 in the passive Facebook use condition, 22 in the active Instagram use condition, 26 in the passive Instagram use condition, 27 in the active Twitter use condition, and 35 in the passive Twitter use condition. The only difference concerns the SNS usage time: participants used the SNS 20 minutes instead of 10 minutes.

#### Post-manipulation measures

We used the same scales as before (Diener et al., 1985; Hofhuis et al., 2019; Lim & Yang, 2019; Thompson, 2007). We asked participants about their satisfaction with life (ω = 0.84), their positive affect (ω = 0.64) and their negative affect (ω = 0.80) after the experimental manipulation. We also measured the general frequency of use of Instagram, Facebook, and Twitter. We finally assessed the perceptions of upward social comparison (ω = 0.85), social support (ω = 0.86), benign envy, and malicious envy on Facebook, Instagram, and Twitter.

In addition, we measured the content that participants saw during their surfing through two questions. The first asked them ‘If you were to summarize the content of this [Facebook][Instagram][Twitter] surfing, which theme(s) would you choose?’ They were able to choose one or more of these topics: everyday life; technology (automotive, high-tech, etc.); politics and/or information; professional; travel, vacation and/or nature; fashion and/or decorative; food and/or sports; brand image and advertising; humoristic; other. We also measured the valence of this content on a seven-point scale (1 = very negative; 7 = very positive). Finally, participants were asked what device they have used to surf on the SNS: computer, smartphone, connected watch, tablet, or other.

### Results

All analyses were conducted on R software version 4.1.3. An exploratory data analysis (EDA) is available on OSF (https://osf.io/9wybs/files/osfstorage/622f40cd46bf100cc4593a72).

#### Preliminary analyses

##### Compliance check

As in Study 1, we measured the time participants spent on the SNS during experimental manipulation. After controlling for age and gender, time of use did not differ regardless of the type of SNS and their usage (for interaction effect, F(2, 150) = 1.135, p = 0.324, η^2^_G_ = 0.015).

##### Surfing content

**Figure 4:**
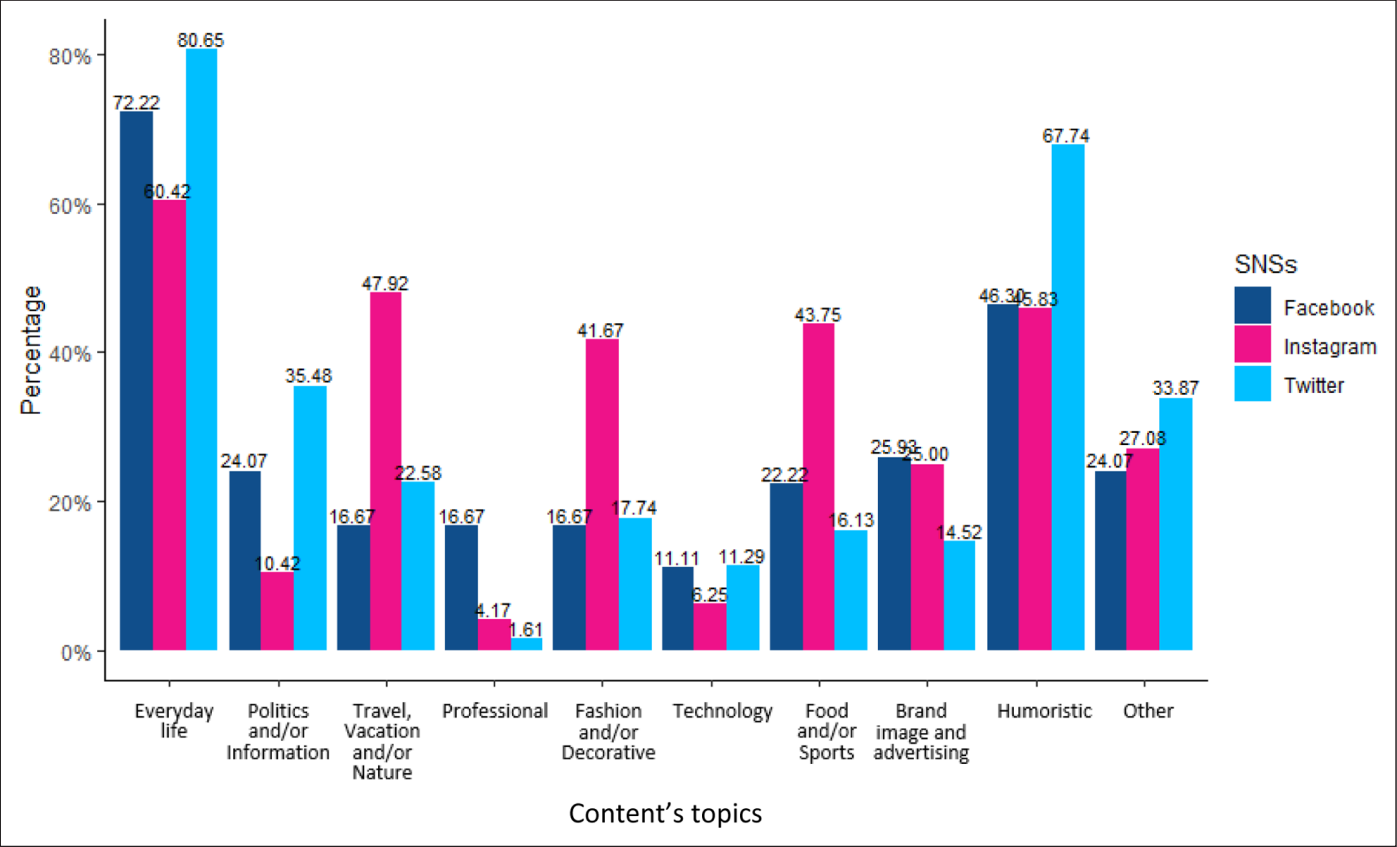
Content topics according to the SNS.

As we can see in Figure 4, content topics were related mainly to everyday life regardless of the SNS (mentioned by more than 60% of the participants). However, we also found descriptive differences between SNSs. For example, content covering travels, vacations, nature, fashion, decoration, food, or sports was mostly present on Instagram (more than 40%). In contrast, Twitter’s content was mostly humoristic (67.74%) and dealt with information or politics (35.48%).

Regarding content valence, surfing on Instagram was perceived as more positive (M = 5.33, SD = 1.17) than surfing on Twitter (M = 4.94, SD = 1.14) or on Facebook (M = 4.85, SD = 1.16). After controlling for age and gender, this difference was not significant (F(2, 153) = 2.518, p = 0.084, η^2^_G_ = 0.032). However, gender had an effect on the perception of the content valence: women perceived the content less positively than men, F(1, 153) = 9.015, p = 0.003, η^2^_G_ = 0.056. This result can be interpreted in two ways: women may have a more negative perception of the content they see on SNSs, or women may not be exposed to the same content as men.

Finally, we looked at the association between content valence and content topics. Multiple linear regression showed that surfing related to political information or politics was negatively associated with content valence (β = –0.560, t(153) = –2.579, p = 0.011). No other significant association was found.

#### Main analyses

For all components of subjective well-being (satisfaction with life, positive affect, and negative affect), we performed a 3 (Type of SNS: Facebook vs. Instagram *vs* Twitter) × 2 (Usage of the SNS: active vs. passive) × 2 (Time: before using SNS vs. after using SNS) repeated measures ANCOVA.^9^ We used the covariates presented above: age, gender,^10^ content valence, content topics,^11^ and device.^12^

##### Satisfaction with life

**Figure 5:**
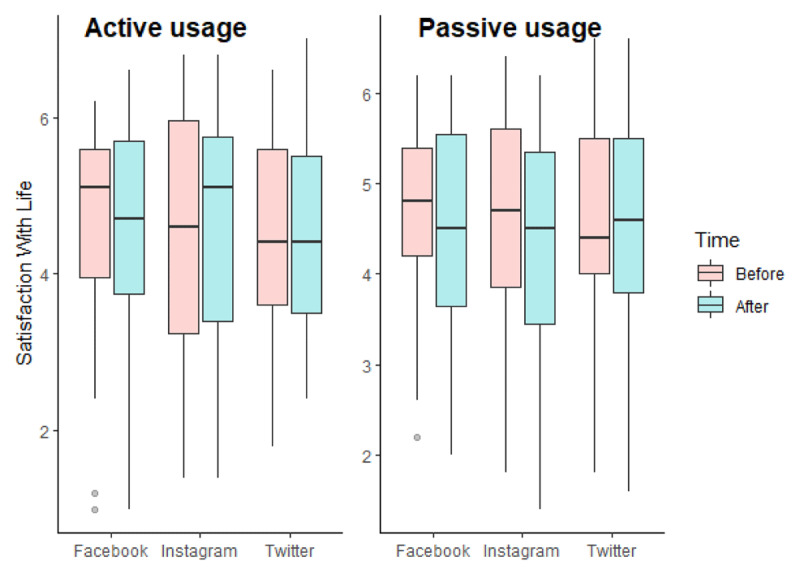
Boxplots of satisfaction with life according to the experimental conditions in Study 2.

Descriptive statistics are shown in Table 8 and a graphical representation is available in Figure 5. The repeated measures ANCOVA for satisfaction with life did not reveal any significant effect of our independent variables (for interaction effect, F(2, 137) = 2.161, p = 0.119, η^2^_G_ = 0.001). We however found an effect of covariates: satisfaction with life was statistically associated with participants’ age (F(1, 137) = 9.991, p = 0.002, η^2^_G_ = 0.066), persons with another gender identity compared to women (F(1, 137) = 4.705, p = 0.032, η^2^_G_ = 0.032) and content valence (F(1, 137) = 16.623, p = 0.000, η^2^_G_ = 0.104). There was a negative association between age and satisfaction with life at time 2, r(158) = –0.226, p = 0.004. In addition, correlation showed that the more positively participants perceived the content on one SNS, the more their satisfaction with life increased at time 2 (r(162) = 0.314, p = 0.000). There was also an interaction effect between time and content valence, F(1, 137) = 9.728, p = 0.002, η^2^_G_ = 0.003. This result means that the difference in satisfaction with life between time 1 and time 2 is associated with the content of the surfing.

**Table 8:** Descriptive statistics for satisfaction with life in Study 2.


SNS	USAGE	T_1_ SWL M(SD)	T_2_ SWL M(SD)

Facebook	Active	4.65 (1.39)	4.63 (1.43)

	Passive	4.66 (1.12)	4.46 (1.24)

Instagram	Active	4.53 (1.56)	4.57 (1.58)

	Passive	4.45 (1.33)	4.30 (1.41)

Twitter	Active	4.56 (1.28)	4.50 (1.27)

	Passive	4.54 (1.15)	4.51 (1.19)


We finally performed a repeated measures ANCOVA with an objective Bayesian approach. However, we have 16 covariates in Study 2. Unlike the NHST approach, the Bayesian approach ‘contrasts the predictive performance of competing models’ (van den Bergh et al., 2020: 80). Doing so, with so many covariates, there are over 600 models to compare. It was, therefore, not possible to compute the results for each model. We decided to remove from covariates the device and the content topics which have no significant impact in the NHST repeated measures ANCOVA. The exclusion Bayes factors for the models including our independent variables are between 1.74 and 12.97, meaning that our data provides anecdotal to substantial evidence in favor of H0 (see Table 9). Consistent with the NHST, the data are more likely under the models including age (BF_excl_ = 0.04), persons with another gender identity compared to women (BF_excl_ = 0.11) and content valence (BF_excl_ = 0.005). In other words, the data are about 200 times (1/0.005) more likely under the models that include the content valence than under the models without it.

**Table 9:** Model-averaged results for satisfaction with life in Study 2.


EFFECTS	BF_EXCL_

Time	1.74

SNS	2.34

Usage	2.24

Age	0.04

Men *vs*. Women	2.89

Another *vs*. Women	0.11

Content’s valence	.005

Time*SNS	12.97

Time*Usage	2.48

SNS*Usage	1.76

Time*SNS*Usage	3.44


##### Positive affect

Descriptive statistics for the positive affect are presented in Table 10.

**Table 10:** Descriptive statistics for positive affect in Study 2.


SNS	USAGE	T_1_ PA M(SD)	T_2_ PA M(SD)

Facebook	Active	3.66 (0.57)	3.57 (0.69)

	Passive	3.42 (0.63)	3.24 (0.65)

Instagram	Active	3.30 (0.66)	3.19 (0.73)

	Passive	3.25 (0.82)	3.08 (0.84)

Twitter	Active	3.22 (0.48)	3.13 (0.70)

	Passive	3.62 (0.55)	3.29 (0.78)


**Figure 6:**
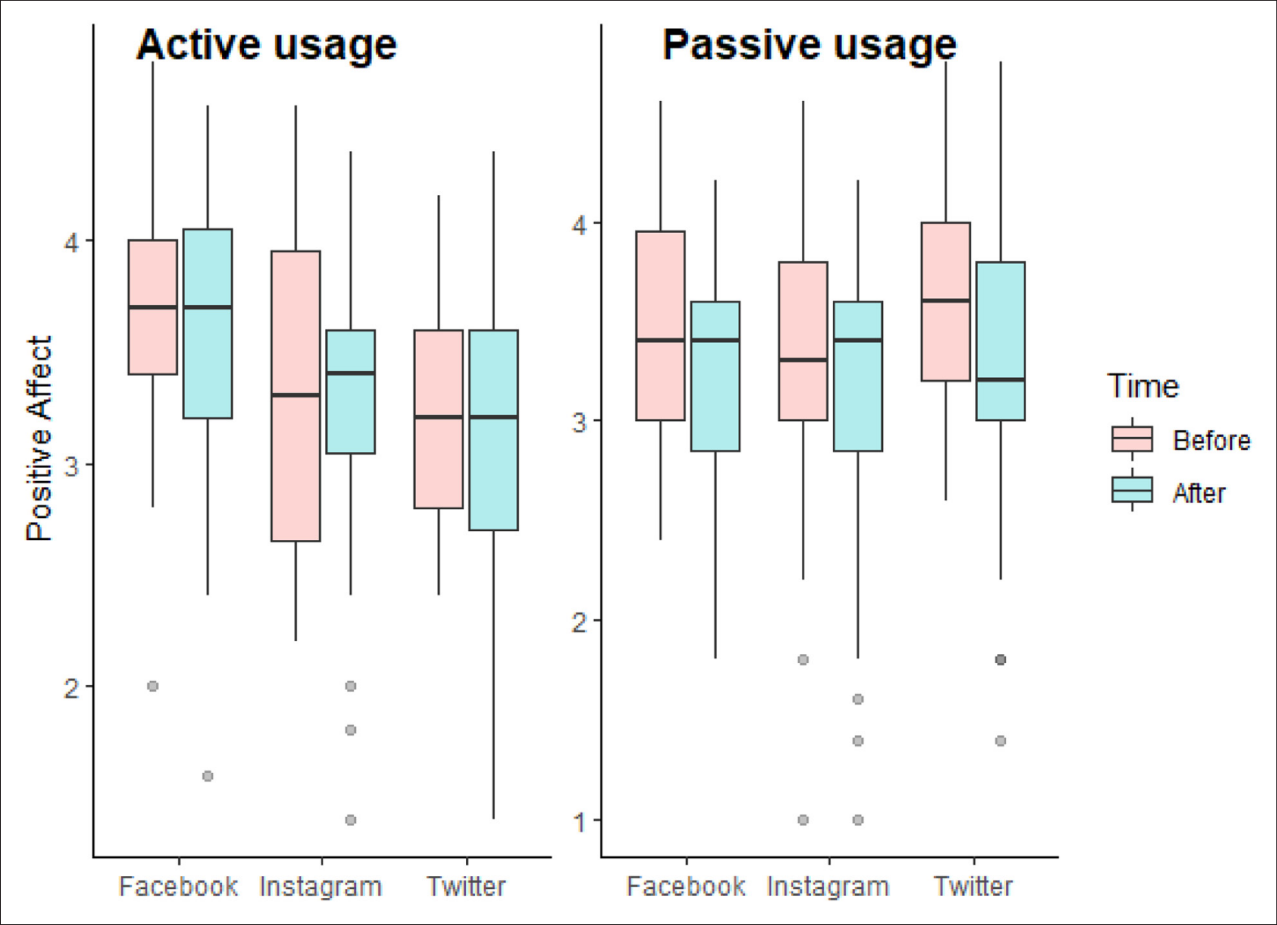
Boxplots of positive affect according to the experimental conditions in Study 2.

As we can see in Figure 6, the repeated measures ANCOVA for positive affect revealed an interaction effect between SNS and usage, F(2, 137) = 3.209, p = 0.043, η^2^_G_ = 0.040. No significant effect of our covariates was found. We performed pairwise comparisons with the Bonferroni correction. Positive affects were higher for active Facebook usage than for active Instagram usage (p.adj = 0.013) and active Twitter usage (p.adj = 0.001). However, as we can see in Table 10, this difference in positive affect within the active conditions seems to be present before the experimental manipulation.

Concerning the objective Bayesian approach, the model-averaged results across matched models are presented in Table 11. The exclusion Bayes factors for the models including usage, content valence, the interaction between time and SNS, the interaction between time and usage, the interaction between time, usage and SNS, and finally, gender, are superior to one. In addition, the data provide no evidence for H0, nor the alternative hypothesis concerning models including the main effect of SNS and age (BF_excl_ ≈ 1). Finally, the exclusion Bayes factors provide evidence in favor of the alternative hypothesis for including the interaction between SNS and usage, and time (BF_excl_ < 1).

**Table 11:** Model-averaged results for positive affect in Study 2.


EFFECTS	BF_EXCL_

Time	0.0003

SNS	0.87

Usage	3.50

Age	1.21

Men *vs* Women	2.53

Another *vs* Women	2.38

Content’s valence	1.87

Time*SNS	10.64

Time*Usage	1.38

SNS*Usage	0.56

Time*SNS*Usage	5.31


##### Negative affect

The descriptive statistics for the negative affect are presented in Table 12.

**Table 12:** Descriptive statistics for negative affect in Study 2.


SNS	USAGE	T_1_ NA M(SD)	T_2_ NA M(SD)

Facebook	Active	2.49 (0.79)	2.26 (0.76)

	Passive	2.37 (0.90)	2.22 (0.76)

Instagram	Active	2.58 (0.74)	2.29 (0.87)

	Passive	2.28 (0.87)	2.27 (0.99)

Twitter	Active	2.31 (0.74)	2.21 (0.81)

	Passive	2.19 (0.81)	2.05 (0.82)


**Figure 7:**
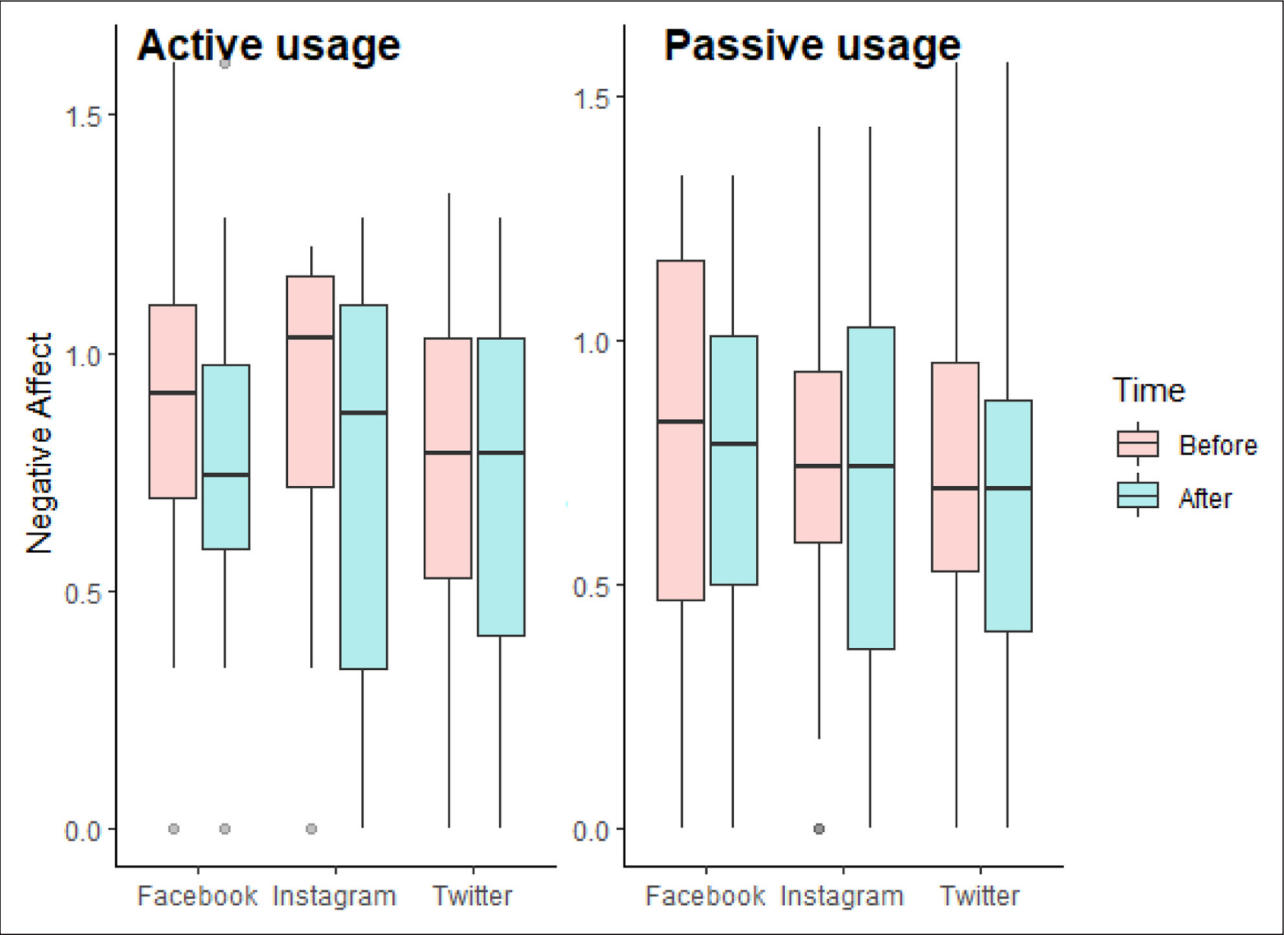
Boxplots of log-transformed negative affect according to the experimental conditions in Study 2.

As we can see in Figure 7, the repeated measures ANCOVA revealed no significant effect of our independent variables on negative affect (for interaction effect, F(2, 137) = 1.099, p = 0.336, η^2^_G_ = 0.001).^13^ We found an effect of the covariates: an interaction effect between men compared to women and time (F(1, 137) = 4.182, p = 0.043, η^2^_G_ = 0.002), an interaction effect between persons with another gender identity compared to women and time (F(1, 137) = 4.858, p = 0.029, η^2^_G_ = 0.003) and content valence (F(1, 137) = 6.837, p = 0.010, η^2^_G_ = 0.044). Correlation showed a negative association between content valence and negative affect at time 2 (r(162) = –0.156, p = 0.047).

We conducted a repeated measures ANCOVA with an objective Bayesian approach (Table 13). The exclusion Bayes factors are between 1.40 and 13.19, except for the models including content valence (BF_excl_ = 0.42) and time (BF_excl_ = 0.002).

**Table 13:** Model-averaged results for negative affect in Study 2.


EFFECTS	BF_EXCL_

Time	0.002

SNS	3.18

Usage	1.40

Age	2.09

Men *vs* Women	1.67

Another *vs* Women	2.01

Content’s valence	0.42

Time*SNS	13.19

Time*Usage	2.57

SNS*Usage	3.02

Time*SNS*Usage	2.81


#### Exploratory analyses

##### Upward social comparison, benign envy, and malicious envy

After controlling for our covariates, one-way ANCOVAs did not reveal any significant effect of the type of SNS on upward social comparison (F(2, 150) = 2.968, p = 0.054, η^2^_G_ = 0.038), benign envy (F(2, 150) = 0.754, p = 0.472, η^2^_G_ = 0.010), and malicious envy (F(2, 150) = 0.621, p = 0.539, η^2^_G_ = 0.008). We, however, found an association between malicious envy and content valence (F(1, 150) = 11.878, p = 0.001, η^2^_G_ = 0.073).

In addition, we found a positive association between upward social comparison and both envies: benign (r(52) = 0.307, p = 0.024 for Facebook; r(60) = 0.264, p = 0.038 for Twitter), and malicious (r(52) = 0.381, p = 0.004 for Facebook; r(46) = 0.320, p = 0.027 for Instagram; r(60) = 0.419, p = 0.001 for Twitter). There was no association between benign envy and malicious envy, except for Twitter (r(60) = 0.429, p = 0.001). There was also no association between the frequency of SNS use and the perception of upward social comparison, benign envy, or malicious envy, on the platform.

Regarding surfing content, a negative association between content valence and malicious envy was found (r(162) = –0.242, p = 0.002). We also regressed our three constructs on the content topics. Results showed that the more participants saw fashion or decorative content during their SNS surfing, the more they perceived upward social comparison (β = 1.058, t(153) = 3.315, p = 0.001). In addition, the more participants saw humoristic content during their SNS surfing, the more they perceived benign envy (β = 0.561, t(153) = 2.171, p = 0.032). Finally, the more participants saw brand image and advertising content during their SNS surfing, the more they perceived malicious envy (β = 0.303, t(153) = 2.411, p = 0.017).

##### Social support

After controlling for our covariates, the one-way ANCOVA revealed an effect of the type of SNS on social support, F(2, 150) = 4.111, p = 0.018, η^2^_G_ = 0.052. Associations with participants’ age (F(1, 150) = 9.994, p = 0.002, η^2^_G_ = 0.062) and content’s valence (F(1, 150) = 5.897, p = 0.016, η^2^_G_ = 0.038) were also found. Bonferroni’s multiple comparison tests showed that social support on Facebook (M = 3.21, SD = 1.60) and Instagram (M = 3.22, SD = 1.48) are less important than social support on Twitter (M = 4.02, SD = 1.35). Finally, there was no association between social support and the frequency of SNS use (p > 0.05).

Regarding the association between social support and surfing content, the more positively the participants perceived the content on one SNS, the more social support they perceived on the platform (r(162) = 0.162, p = 0.038). In addition, surfing related to everyday life was positively associated with social support (β = 0.567, t(153) = 2.124, p = 0.035). We did not find any other significant association.^14^

### Brief discussion

Study 2’s results are in line with our previous findings: neither the modalities of use nor the type of SNS significantly influenced subjective well-being (Masciantonio et al., 2021; Verduyn et al., 2017). Again, our hypotheses cannot be confirmed, but Bayesian analyses most often suggest only anecdotal evidence in favor of H0 regarding the effect of passive and active usages. In addition, although we controlled for the device used to navigate SNSs, we did not find significant effects. This study provides, however, an interesting contribution regarding SNSs surfing content.

First of all, the difference in surfing content across SNSs was consistent with the literature: Instagram content was perceived more positively than Facebook and Twitter contents (Waterloo et al., 2018), Twitter topics concerned information and politics (Johnson & Yang, 2009), and Instagram topics were related to popularity and creativity (Sheldon & Bryant, 2016). Twitter use was also mainly related to humoristic content (e.g., memes or GIFs). More significantly, our results showed that the valence of the content is associated with satisfaction with life and negative affect: the more positive the SNSs’ content, the more satisfaction with life increased and negative affect decreased. The psychological processes—upward social comparison, envy, and social support—explaining in the literature the relation between SNS usage and subjective well-being are also related to the SNSs’ surfing content. For example, publications related to everyday life were positively associated with social support. On the contrary, those about brand image and advertising were positively associated with malicious envy. In the same vein, publications referring to fashion and decoration were positively associated with upward social comparison. Finally, humoristic publications were positively associated with benign envy. These results therefore provide important research perspectives with regard to the effects of SNSs on subjective well-being.

Regarding the SNSs comparative approach, our results are partially in line with those of Study 1. We found that the perception of upward social comparison, benign envy, and malicious envy did not differ depending on the platform. Upward social comparison was associated with both envies, but only for Facebook and Twitter. Benign envy and malicious envy were also linked only for the Twitter platform. Finally, social support was perceived as more present on Twitter than on Instagram and Facebook. This latter result is inconsistent with Study 1; however, the use of Facebook among youth appears to be motivated by social pressure rather than peer interaction (Masciantonio & Bourguignon, 2020). Pioneering work showed, for example, that younger people tend to assign different meanings to SNSs than older persons (Boczkowski et al., 2018).

This study is not devoid of limitations. Firstly, although the sample was consistent with the literature on SNSs, it was composed of French students, mostly women, and gender and age have central importance on how SNSs are used. Indeed, a recent study shows that the relationship between social media and life satisfaction was more negative for girls than for boys and was dependent on age-related developmental windows (Orben et al., 2022). In Study 1, age is positively associated with life satisfaction, whereas the opposite is observed in Study 2. It should be noted, however, that age does not have a linear relationship with well-being. The latter decreases from the age of 20 until 50 years old, then it increases again from 50 years old until its highest point, between 65 and 70 years old, then it decreases again (Afsa & Marcus, 2008). Secondly, the SNS usage time being longer, the number of participants is smaller than Study 1. Thirdly, we did not find a significant effect for the type of device, but most of our participants used their smartphones to take part in the study.

## General Discussion

This research aimed to investigate the differential effects of passive and active uses of Facebook, Instagram, and Twitter on subjective well-being. In doing so, we tried to respond to some of the limitations of the literature by conducting two preregistered experimental studies. Our results do not reveal a significant effect of SNSs on subjective well-being, regardless of the modalities of usage or the type of SNS. However, while some Bayesian analyses are strongly in favor of H0, others offer rather anecdotal evidence. This suggests that further investigation of this research question has to be done in diverse and larger samples, but also that other factors need to be considered. The literature abounds of possible avenues of research, including, for example, users’ motivations (Sun & Zhang, 2021). This research, for its part, opens the reflection on SNSs surfing content. We found that the more people see positive content on SNSs, the more satisfied they are with their lives and the less they feel negative emotions.

These results are in line with recent literature focusing on the SNSs surfing content (Choi & Kim, 2020; Meier & Reinecke, 2021; Valkenburg, 2022). This is also in line with the Extended Active-Passive Model, which emphasizes the content that users are confronted with during their SNSs navigation (Verduyn et al., 2022). People can experience a lot of interactions and see a plurality of publications on SNSs: this complexity may not be captured by focusing only on specific usages or specific platforms. If someone actively uses SNSs but receives hate messages, it seems unlikely that this would lead to positive affect. Likewise, passively using SNSs to find new creative ideas may have positive effects on their well-being. Thus, considering the surfing content seems to be an interesting line of research as the content that users are exposed to is eventually a reflection of the differences between SNSs and the ways people use them. For example, Twitter is the SNS dedicated to news and politics (Park, 2013). Yet, this content is known to lead to symptoms of anxiety (Bodas et al., 2015). If we consider only the cross-media perspective, we would, therefore, expect that Twitter undermines mental health compared to more positive SNSs like Instagram. However, how individuals use Twitter may qualify this conclusion: if Twitter is used to caricature politics through the sharing and production of memes, then one can assume that this type of active use may rather improve mental health. Doing so, considering the SNSs surfing content in addition to the type of SNS and the modalities of usage could fit more realistically with the actual way SNSs invaded our lives. This, we believe, may be part of the reason why we cannot validate our hypotheses: knowing whether and how an individual decides to use a particular SNS is not enough to capture the experience they will live, the content of this interaction is determining.

Although this research fills the gap in the literature in many ways, it is not devoid of limitations, both methodological and theoretical. First, our operationalization of passive and active usages combines both private and public activities. One might hypothesize that private and public uses of SNS may have different effects on subjective well-being, notably because their frequency and nature are different (Valkenburg et al., 2021). It is also worth noting that users rarely use SNSs only actively, most of the time they combine passive and active use; and at least some passive usage is required to be able to use SNSs in an active way, making it more difficult to concretely disentangle the two (Gerson et al., 2017). In addition, we have chosen to conduct an experimental exposure. This method avoids the constraints of self-reported scales, but the effects of SNSs on subjective well-being may occur after repeated exposures (Appel et al., 2016). Similarly, it is possible that the exposure time was too short to affect subjective well-being, or that it could only affect some of its components. Indeed, positive and negative affect are sometimes considered a hedonic form of well-being, whereas satisfaction with life would be more of a eudaimonic form (Deci & Ryan, 2008). It is also possible that the relatively brief exposure time caused participants to question the objectives of the studies, which we did not verify with a control question. Moreover, we decided to treat each SNS one by one, whereas users continuously navigate between several platforms (platform-swinging; Tandoc et al., 2019). Furthermore, the literature has recently highlighted the need to study person-specific media effects (Beyens et al., 2020). Only a small subset of individuals would have a negative experience with SNSs, while for the vast majority, SNSs would have no consequences or positive ones. It should equally be taken into account that we had little way to verify participant compliance, and that other methods such as screen recording will need to be implemented in future studies (Verduyn et al., 2015). Last but not least, the effect size of SNSs may have been overestimated in the literature (Orben & Przybylski, 2019), which means that our sample size might have been too small. In the same way, the literature assumes that SNSs use impacts subjective well-being, but this relationship could also be nonlinear, inverse, or bidirectional.

Therefore, we encourage researchers to pursue the reflections on the type of SNS, the modalities of usage, the device used, and the surfing content. On this last concept, many questions are still pending: what characteristics of the content matter (valence, intensity, topic, ambiguity, etc.)? Do we need to consider the whole message rather than specifically the content (Meier & Reinecke, 2021)? How are passive and active usages associated with content? Are certain SNSs more conducive to certain content? What psychological processes are at stake (Kross et al., 2021)? This research already gives us some insights: valence seems to be more important than the topic and to be more positive for some SNS. Valence and topics are also related to well-known psychological processes (social support, envy, and social comparison). But these results are only a preliminary conclusion and further research will be required.

## Conclusion

This research is the first to experimentally investigate the effects of different SNSs on subjective well-being by considering how they are used. Two studies were conducted on student and non-student samples, controlling for numerous covariates such as the device used and the surfing content. Subjective well-being was also conceptualized through its two components, cognitive and affective. In doing so, this research integrates a large part of the literature, while overcoming some of its limitations including the use of cross-sectional designs, the focus on Facebook, and the absence of consideration for the active-passive usages. Our results did not demonstrate differential effects of active and passive uses of Facebook, Twitter, and Instagram on subjective well-being. The major result of this research was rather the association found between surfing content and subjective well-being: positive content is associated with more life satisfaction and fewer negative emotions. However, no single study can claim to be generalizable, and this study is no exception. Therefore, this conclusion does not mean that we should consider SNSs as a homogeneous media, or that we should stop investigating how people use them. Instead, this research provides insights for developing a more integrative and holistic view of the presumed association between SNS use and subjective well-being.

## Open Practices

- Data, materials, and scripts are available at: https://doi.org/10.17605/OSF.IO/9WYBS
- Preregistration for Study 1 is available at: https://doi.org/10.17605/OSF.IO/SE73R
- Preregistration for Study 2 is available at: https://doi.org/10.17605/OSF.IO/S8UXP

## Footnotes

The complete detail of the original data processing is available on OSF (https://osf.io/9wybs/files/osfstorage/621f881e295ed101d82814a4). The high attrition rate can be explained by an inadvertent error on the Prolific platform that led to many participants assigned to the study not owning the three SNSs. As a result, participants were unable to complete the questionnaire. The problem was quickly resolved, and the study was then able to take place normally. For the sake of transparency, we wanted to indicate all the people who accessed the questionnaire.

The correlation and Bayesian analyses are exploratory analyses, they have, therefore, not been preregistered.

We have preregistered that for all statistical analyses we will use gender and age as covariates, and if these variables have no impact, we will redo the analyses by removing them. We, therefore, did the analyses with and without them (all analyses are available on OSF, https://osf.io/9wybs/files/osfstorage/621f881e295ed101d6281a73). Here, we decided to present the analyses with these covariates because they are significantly associated with our dependent variables.

In addition, a non pre-registered MANCOVA with repeated measures was conducted, with the same covariates, to limit the joint error rate and to achieve greater statistical power. The analyses did not show significant within-subject (Wilks’ Lambda, Fs < 1.600, ps > 0.200), or between-subject effects (Wilks’ Lambda, Fs < 1.900, ps > 0.100) of our independent variables on the three dependent variables.

We also conducted exploratory analyses of the relationship between subjective well-being and the variables of social comparison, envy, and social support. Our results are in line with the literature. For example, in line with Appel et al. (2016), the perception of upward social comparison on Facebook (r(78) = –0.250, p = 0.025) and Twitter (r(78) = –0.240, p = 0.032) was negatively associated with life satisfaction at time 2. The association was negative but not significant for Instagram (r(82) = –0.183, p = 0.097). For interested readers, all of these additional analyses are available on OSF: https://doi.org/10.17605/OSF.IO/9WYBS.

The complete detail of the original data processing is available on OSF (https://osf.io/9wybs/files/osfstorage/621f881f295ed101d9281c14). The students gave their verbal agreement to participate in the study during the psychology lectures, but many of them did not finally complete the study at home, despite two email reminders. It should be noted that the study is time-consuming and requires participants to make considerable effort to follow the experimental protocol.

As stated in the preregistration, we stopped data collection ‘once all university student volunteers have completed the questionnaire.’ Unfortunately, we had fewer volunteers than expected, so our sample size is smaller than the power analysis we performed. The sensitivity analysis shows, however, that the study can detect a small to medium effect size.

Doing so, the study was conducted in French.

As in Study 1, we also conducted a repeated measures MANCOVA (with the same covariates as the ANCOVAs). The analyses did not show significant within-subject (Wilks’ Lambda, Fs < 2.300, ps > 0.080) or between-subject effects (Wilks’ Lambda, Fs < 1.500, ps > 0.150) of our independent variables on the three dependent variables.

Gender is a nominal variable with three modalities: woman, man, and another gender identity. We created two dummy variables with ‘woman’ as the reference category.

Participants could choose one or more content topics among the 10 proposed. We therefore created 10 binary variables, one for each topic (0 = absent, 1 = present).

Participants used only three devices: smartphone, computer, or tablet. We created two dummy variables with ‘smartphone’ as the reference category.

The distribution for negative affect was highly right-skewed, and we log-transformed the data. Apart from our main dependent variables, we log-transformed the variables age and malicious envy which were also right-skewed. These transformations had not been anticipated, and therefore they were not preregistered.

Similar to Study 1, exploratory analyses of the relationship between subjective well-being and the social comparison, envy, and social support variables are available on OSF: https://doi.org/10.17605/OSF.IO/9WYBS.
